# Open bite malocclusion and orofacial dysfunction in patients with rare diseases

**DOI:** 10.1093/ejo/cjag037

**Published:** 2026-06-08

**Authors:** Christina Havner, Åsa Mogren, Lotta Sjögreen, Anna Westerlund

**Affiliations:** Department of Orthodontics, Institute of Odontology, The Sahlgrenska Academy, University of Gothenburg, Gothenburg, PO Box 450, Gothenburg SE-405 30, Sweden; Mun-H-Center, Orofacial Resource Center for Rare Diseases, Public Dental Service, Vastra Gotalandsregionen, PO Box 7163 SE-402 33, Gothenburg, Sweden; Mun-H-Center, Orofacial Resource Center for Rare Diseases, Public Dental Service, Vastra Gotalandsregionen, PO Box 7163 SE-402 33, Gothenburg, Sweden; Speech and Language Pathology Unit, Department of Health and Rehabilitation, Institute of Neuroscience and Physiology, The Sahlgrenska Academy, University of Gothenburg, PO Box 455, SE-405 30 Gothenburg, Sweden; Mun-H-Center, Orofacial Resource Center for Rare Diseases, Public Dental Service, Vastra Gotalandsregionen, PO Box 7163 SE-402 33, Gothenburg, Sweden; Speech and Language Pathology Unit, Department of Health and Rehabilitation, Institute of Neuroscience and Physiology, The Sahlgrenska Academy, University of Gothenburg, PO Box 455, SE-405 30 Gothenburg, Sweden; Department of Orthodontics, Institute of Odontology, The Sahlgrenska Academy, University of Gothenburg, Gothenburg, PO Box 450, Gothenburg SE-405 30, Sweden; Mun-H-Center, Orofacial Resource Center for Rare Diseases, Public Dental Service, Vastra Gotalandsregionen, PO Box 7163 SE-402 33, Gothenburg, Sweden

**Keywords:** orthodontics, open bite, rare diseases, neuromuscular diseases, craniofacial abnormalities, macroglossia, muscular hypotonia

## Abstract

**Aim:**

To compare orofacial dysfunctions (OFD) in individuals with rare diseases, with and without open bite (OB) malocclusion, and to explore the associated symptoms.

**Patients and Methods:**

In total, data for 788 individuals representing 164 different rare diseases, were collected from the MHC database for the period of 2013–2019. The inclusion criteria were having a rare disease and having completed the Nordic Orofacial Test—Screening (NOT-S). OB was categorized as anterior OB (AOB), lateral OB (LOB), and severe anterior OB (AOB_S_). The sample was divided into two groups: 142 with OB (mean age, 14.2 ± 13.0 years; 81 males, 61 females); and 557 with normal vertical relation (NVR) (mean age, 15.1 ± 12.9 years; 292 males, 265 females). OFD was compared between the groups. Items from the NOT-S test were analysed, and the Odds Ratio (OR) for OB was calculated.

**Results:**

The OB prevalence was 18%, with AOB being the most-common sub-type (58%), followed by AOB_S_ (15%) and LOB (13%). Nemaline myopathy was the disease with the highest prevalence rates for OB and AOB_S_. The OB group had a larger proportion of OFD than the NVR group in 6/12 domains of the NOT-S. Deviant tongue posture was most strongly associated with OB (OR 6.062).

**Conclusion:**

Two-thirds of the individuals had OFD, and OB was a common finding (18%). Rare diseases with symptoms of orofacial hypotonia, craniofacial abnormalities and macroglossia show a higher prevalence of OB. A deviant tongue posture showed the highest odds for OB in this group of rare diseases.

## Introduction

Open bites (OB) are among the most challenging malocclusions to treat. This is because the aetiology of OB is multifactorial and is often associated with orofacial dysfunctions (OFD), such as oral habits, open mouth posture and anterior tongue position [1–3]. Although the treatment may initially show success [4], the risk of orthodontic relapse remains significant if the underlying dysfunction is not resolved [5]. Many different orthodontic treatment approaches have been used to correct OB in both growing and nongrowing individuals. There is still no conclusive evidence of the effectiveness of specific treatment interventions or appliances [6–9].

To achieve a long-term, positive treatment outcome, it is essential to understand the underlying cause of the OB in order to address it appropriately. The inter-dependence of ‘form and function’ is a well-established concept in biology, although there are different schools of thought regarding cause and effect [10].

A group with an increased tendency to have OB and OFD includes patients with rare diseases [11]. While rare diseases affect <5 in 10 000 individuals [12], collectively 6%–8% of the population are estimated to suffer from a rare disease in Europe. This often means that the affected individuals experience complex and disabling symptoms that are chronic and sometimes life-threatening. It has been reported that almost 50% of these individuals display OFD, such as problems with feeding or eating, swallowing, speech and saliva control [13]. In most cases, rare diseases display overlapping symptoms of growth disturbances, cognitive disabilities and neuromuscular impairments that originate from both the central and peripheral nervous systems or the muscle itself. These symptoms often share the same genetic background [14].

Dental malocclusions are also more common in individuals with rare disease. OB is one of the most-common dental malocclusions in patients with OFD and rare diseases [11], as well as in patients with intellectual disabilities [15]. Severe malocclusion may have negative effects on chewing efficiently and swallowing safely [16].

An OB can be defined as the loss of vertical contact between antagonizing teeth in one segment and can be categorized as an anterior OB (AOB) or a lateral OB (LOB). The prevalence of OB in Swedish school children is 4% [17], whereas the global prevalence of anterior OB in children and adolescents is 16.5% (age range, 2–16 years) [18]. In the global adult population, OB has been identified in 4%, with a higher prevalence found in the African population [19]. The prevalence rates for groups with intellectual disabilities, as well for those with rare diseases (and more specifically, neuromuscular diseases) have been reported as ∼40%–60% [11, 15, 20, 21].

By analysing the prevalence of OB malocclusions in a population with rare diseases and comparing the functional profiles of patients with and without OB, we can gain valuable insights into the factors associated with OB and, thereby, improve our overall treatment approach.

## Subjects and methods

The study sample was collected from the MHC database (Mun-H-Center, Gothenburg, Sweden) for the period of 2013–2019. The inclusion criteria were individuals ≥3 years of age with a rare disease (affecting <5 in 10 000) and having completed an OFD screening test.

### Subjects

All of the subjects were recruited upon referral to Mun-H-Center, a national orofacial resource centre for rare diseases in Sweden, or when attending the Ågrenska family stay programmes or other clinical research projects. Overall, 788 individuals aged 3–83 years (mean age, 14.5 ± 12.8 years) with 164 different rare diseases were included in the sample. The majority of the subjects were children aged <18 years (*N* = 636).

**Figure 1:**
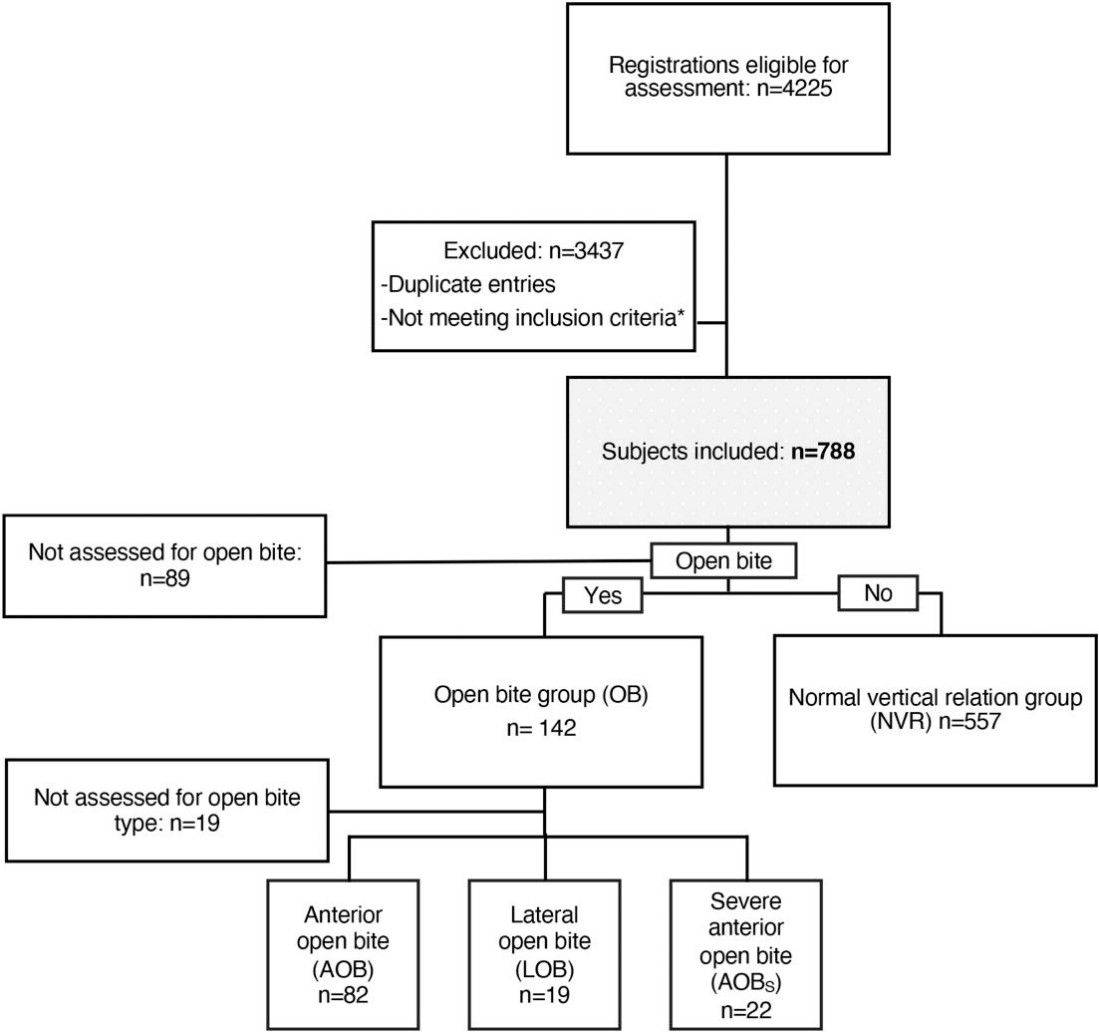
The flowchart illustrates the flow of participant registrations from the MHC database that were included in the study as well as group allocation. Following eligibility assessment, participants were divided into two groups: one with open bite (OB) and one with normal vertical occlusal relationship (NVR). The open bite group was further categorized into three subtypes: lateral open bite (LOB), anterior open bite (AOB), and severe anterior open bite with occluding contacts only on molars (AOB_S_). * *Completing the Nordic Orofacial Test—Screening was one of the inclusion criteria and this test was not included in the assessment protocol for the database until 2013. This was the primary reason for exclusion in the study.*

The sample was divided into two groups: a group that included 142 subjects with OB (mean age, 14.2 ± 13.0 years; 81 males, 61 females); and a group that had 557 individuals with normal vertical relation (NVR) (mean age, 15.1 ± 12.9 years; 292 males, 265 females). There were 89 subjects who were not assessed for OB in the sample (Fig. 1).

To detect differences in the prevalence rates of OB at different ages, the subjects were also divided into five age groups (Table 1).

**Table 1:** Age-group distribution and open bite prevalence.

Group	1	2	3	4	5
**Age (years)**	3–9	10–18	19–30	31–50	51–83
***N***	374	277	57	58	22
**OB (%)**	18%	15%	25%	25%	23%

The sample was divided into five age groups. The majority of participants (83%) were in age groups 1 and 2. The prevalence of open bite (OB) in the different age groups varied between 15% and 25%.

Thirty-eight of the participants had received some type of orthodontic treatment of which 3 also were treated in combination with orthognathic surgery. In the age groups 3, 4, and 5 (age 19–83, *n* = 127) 25 (20%) individuals had received orthodontic treatment.

There were 44 individuals diagnosed with cleft palate and/or cleft lip, of which some had undergone surgical closure of the soft palate, and some also closure of the hard palate and bone grafting procedures that may inhibit transversal and sagittal growth. Among them, 10 individuals also had a genetic craniofacial syndrome associated with deficient facial growth.

### Methods

#### Clinical examination

The participants were examined in a dental clinical setting in either a dental chair or a wheelchair. All the assessments were made by a dentist and a speech-language pathologist working together. For this sample, 13 dentists and 6 speech-language pathologists collected the data.

Data were collected using the MHC Orofacial Observation Chart [11, 21], together with the Nordic Orofacial Test—Screening (NOT-S) tool [22]. The MHC observation chart and the NOT-S have a complete manual for definitions and guidance (see supplementary). The NOT-S was used to assess co-existing OFD and is a validated and reliable test. All of the examiners were introduced to the test, instructed on how to make the measurements, and were calibrated before the start of the tests. The examiners attended the calibration meetings held annually at Mun-H-Center.

#### Questionnaire

The NOT-S also contains an interview part. Care-givers assisted the participants during the interview part when needed. The maximum score on the test is 12 points, 1 point for each domain and 6 points for each part. A total test score >2 reflects OFD [23].

#### Variables

##### Primary outcome variable

OB was the primary outcome and was defined as an intermaxillary distance of >0 mm in the anterior or lateral or both segments. A segment was defined as at least 3 pairs of adjacent antagonists. Depending on the location of the OB, it was categorized as AOB, LOB or severe anterior OB (AOB_S_). AOB_S_ meant having only occlusal contacts on one or more pair of molars. Teeth undergoing eruption and primary teeth intra-occluded due to ankylosis were not considered as OB.

##### Secondary outcome variables

The domains of the interview part of the NOT-S were (I–VI): *Sensory*, *Breathing*, *Habits*, *Chewing and swallowing*, *Drooling*, and *Dry mouth*. The domains of the examination part were (1–6): *Face at rest*; *Nose breathing*; *Facial expression*; *Masticatory muscles and jaw function*; *Oral motor function*; and *Speech*. Each domain contained one or more items (listed in Table 2).

**Table 2:** Orofacial dysfunction by test item and association with open bite (OR).

Interview part, NOT-S, all items	Tot. sample (*n* = 788)	OB	NVR	OR	95% CI lower	95% CI higher
***I Sensory***						
Gag reflex when brushing	14%	13%	12%	1054	0.648	1.714
Too much food to chew	13%	13%	12%	1.07	0.657	1.742
***II Breathing***						
Breathing support	5%	13%	3%	4.14	2.232	7.68
Snoring/Apnoea	21%	25%	19%	1.332	0.958	1.853
***III Oral habits***						
Nail biting, finger sucking daily	12%	13%	10%	1.217	0.742	1.998
Lip biting, tongue/cheek sucking daily	23%	23%	21%	1.097	0.781	1,54
Clenching or grinding teeth daily	15%	11%	14%	0.774	0.459	1.305
***IV Chewing and swallowing***						
No oral feeding	11%	13%	10%	1.406	0.861	2.297
Avoiding certain consistencies	31%	35%	29%	1.179	0.908	1.531
Meal takes >30 mins	21%	23%	20%	1.156	0.821	1.627
Swallows large bites	16%	13%	16%	0.837	0.529	1.326
Coughs during meals	8%	10%	6%	1.615	0.891	2.927
***V Drooling***						
Saliva on chin daily	24%	34%	20%	1.652	1.245	2.191
Needs water to eat cracker	15%	11%	17%	0.661	0.402	1.085
Sore mouth/tongue	2%	1%	2%	0.654	0.148	2.888
**Examination part, NOT-S all items**						
***1 Face at rest***						
Asymmetry	9%	11%	9%	1.255	0.737	2.137
Deviant lip posture	30%	41%	29%	1.422	1.122	1.803
Deviant tongue position	4%	12%	2%	6.062	2.905	12.651
Involuntary movements	2%	2%	2%	1.177	0.328	4.22
***2 Nose breathing***						
Nose breathing	7%	13%	6%	2.017	1.178	3.454
***3 Facial expressions***						
Eyes closed tightly	21%	23%	21%	1.064	0.754	1.501
Show teeth	12%	12%	13%	0.926	0.564	1.52
Whistle/blow	15%	16%	15%	1.061	0.696	1.619
***4 Masticatory and jaw function***						
Bite hard on back teeth	17%	27%	15%	1.821	1.307	2.538
Open mouth wide	8%	18%	6%	3.09	1.913	4.993
***5 Oral motor***						
Stick your tongue out	6%	10%	6%	1.664	0.916	3.024
Lick your lips	16%	22%	15%	1.431	0.99	2.066
Blow up cheeks	13%	17%	13%	1.29	0.845	1.037
Soft palate elevation	6%	8%	6%	1.345	0.717	2.523
***6 Speech***						
No speech	2%	4%	2%	1.961	0.749	5.135
Count to ten	32%	35%	32%	1.108	0.859	1.429
Say ‘pataka’	24%	24%	25%	0.959	0.692	1.33

The table shows the proportion of item-specific dysfunction in the open bite (OB) and normal vertical relation (NVR) groups and the corresponding odds ratios (ORs) for open bite (OB).

### Statistical analysis

The data were analysed using the Statistical Package for the Social Sciences (SPSS Statistics 22). Statistical nonparametric tests (Chi-square) were performed to examine the relationship between OB and OFD. Associations with OB were examined by calculating odds ratios (OR), which were used as measures of association between specific OFD items and the presence of open bite. A binary logistic regression analysis was performed to examine the association between age and OB (binary outcome). Age was included as a continuous predictor, and odds ratios (OR) with 95% confidence intervals (CI) were calculated.

### Reliability tests

Krippendorff’s alpha (α) coefficient [24] was used to estimate the inter-rater reliability of the variables from the MHC chart. Data were used from the assessments of 16 patients (not part of this sample) that were carried out by 8 of the 13 dentists. The occlusal variables demonstrated strong inter-rater reliability (open bite: α = 1.000; Angle Class I: α = 0.894; Angle Class II: α = 1.000; Angle Class III: α = 0.845). The orofacial variable showed moderate inter-rater reliability (lip hypotonia: α = 0.743).

### Ethical considerations

The study was approved by the Swedish Ethical Review Authority (Dnr. 2024-08132-02, 189-95). All of the participants or their parents provided a signed informed consent form.

To ensure that the data were kept anonymized, the prevalence of OB and its sub-types are only displayed in Table 3 if there were nine or more subjects with the same disease.

**Table 3:** Prevalence of open bite in rare diseases.

Diagnosis	N	OB	LOB	AOB	AOB_s_	Type not specified
Duchenne muscular dystrophy	63	37%	11%	16%	8%	2%
Ehlers-Danlos syndrome	54	4%	2%	0%	2%	0%
Myotonic dystrophy type 1	42	24%	0%	19%	5%	0%
22q11 deletion syndrome	34	6%	0%	3%	3%	0%
Williams syndrome	31	19%	0%	13%	3%	3%
Spinal muscular atrophy type 1, 2, 3	27	33%	19%	7%	7%	0%
Marfan syndrome	16	13%	6%	6%	0%	0%
Arthrogryposis Multiplex Congenita	15	20%	0%	7%	13%	0%
Neurofibromatosis type 1	15	7%	0%	7%	0%	0%
Ectodermal dysplasia	14	7%	0%	7%	0%	0%
Tuberous sclerosis	14	21%	0%	21%	0%	0%
Silver-Russel syndrome	13	0%	0%	0%	0%	0%
Limb-Girdle muscular dystrophy	12	42%	8%	8%	17%	8%
Potocki-Lupski syndrome	12	0%	0%	0%	0%	0%
Turner syndrome	12	42%	8%	25%	0%	0%
Huntington’s disease	11	0%	0%	0%	0%	0%
Charcot-Marie-Tooth disease	10	30%	0%	30%	0%	0%
Galactosaemia	10	10%	0%	0%	10%	0%
Nemaline myopathy	10	60%	0%	10%	40%	10%
Apert syndrome	9	56%	0%	44%	0%	11%
Beckwith-Wiedemann syndrome	9	44%	0%	33%	0%	11%
Congenital itchyosis	9	0%	0%	0%	0%	0%
CHARGE syndrome	9	11%	0%	11%	0%	0%
Lawrence-Moon-Bardet-Biedl syndrome	9	0%	0%	0%	0%	0%
Möbius syndrome	9	0%	0%	0%	0%	0%
Prader-Willi syndrome	9	0%	0%	0%	0%	0%
Sotos syndrome	9	33%	0%	33%	0%	0%

The most-common rare diseases in the sample and the prevalence rates of open bite (OB), lateral open bite (LOB), anterior open bite (AOB), severe anterior open bite with antagonist molar contacts only (AOB_S_) in each syndrome.

## Results

### Prevalence of open bite

The prevalence of OB in the sample was 18% (*N* = 142, 81 male, 61 female). AOB was the most-common sub-type (58%), followed by AOB_S_ (15%) and LOB (13%).

When dividing the OB group according to age (Groups 1 to 5), the prevalence ranged from 15%–25% (Table 1). The logistic regression analysis showed that age was not significantly associated with OB (OR 1.01, 95% CI 0.99–1.02, *P* = 0.507). The model demonstrated very low explanatory power (Nagelkerke *R*^2^ = 0.001) indicating that age alone explained only a very small proportion of the variation in OB. In the youngest age group (*N* = 374), 18% had OB and 12% had a non-nutritive sucking habit (mainly digit sucking). Oral habits were observed at a comparable frequency in the NVR group and the OB group within the total sample (Table 2). Among the youngest individuals (at 3–6 years of age, *n* = 202), 21% had a non-nutritive sucking habit, of which 15% had developed an OB.

Of those individuals who displayed AOB_S_, 81% had a neuromuscular disease. The LOB sub-type was presented mainly by individuals with neuromuscular diseases (68%) and was frequently observed (19%) in individuals with the combined forms of spinal muscular atrophy (Table 3). Nemaline myopathy had the highest prevalence rates of OB malocclusion (60%) and of the most-severe sub-type AOB_S_ (40%). The prevalence rates of OB differed between different diseases in the sample; OB was a common finding in groups with orofacial hypotonia (25%), craniofacial abnormalities (38%), and diseases associated with macroglossia (39%). Diseases with orofacial hypotonia originating from the peripheral nervous system (PNS) were more associated to OB both in prevalence and severity, than cases of hypotonia arising from the central nervous system (CNS) in this sample (Table 3).

Posterior crossbite was the most-prevalent co-existing malocclusion and was seen in 38% (35/91), followed by moderate to severe crowding in 27% (34/128), and spacing in 26% (34/132). The majority of the subjects with OB had a normal sagittal relation (41%, 44/102). Class III relation was more common (20%, 22/109) than Class II relation (12%, 13/107) among those assessed in the OB group. Enlarged tonsils (according to Friedman index size 3–4) were seen in 9% (7/75) individuals in the OB group. Among those who reported to have received orthodontic treatment (*n* = 38), 24% presented with an open bite at the time of the assessment.

### Orofacial dysfunction profiles

The total NOT-S score ranged from 0 to 10 points in the sample and 58% had a score of 3 points or more. The subjects with the highest total NOT-S score (≥8 points) had a mean age of 11.5 years (3–46 years), 23% had neuromuscular diseases, and 11% had craniofacial abnormalities.

**Figure 2:**
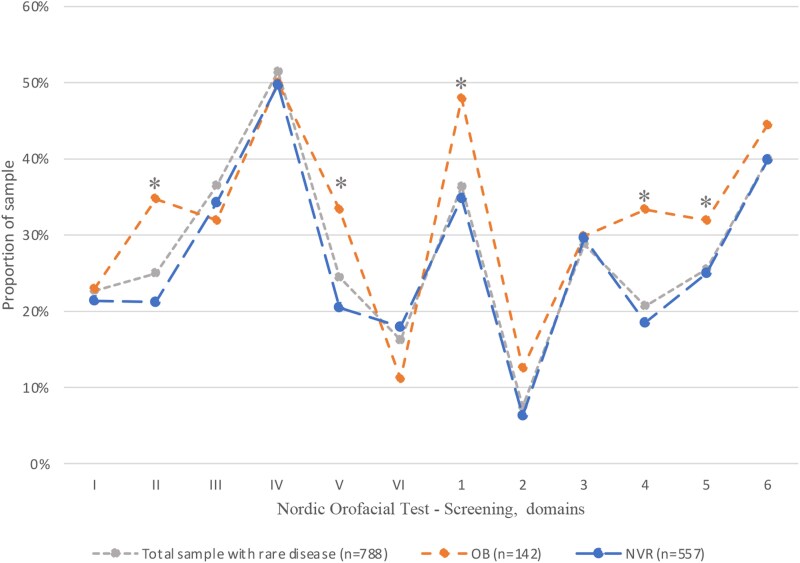
Orofacial dysfunction profiles of patients with rare diseases with open bite (OB) and with normal vertical relation (NVR) using the nordic orofacial test—screening. The domains on the *x*-axis are: I, *Sensory*; II, *Breathing*; III, *Habits*; IV, *Chewing and swallowing*; V, *Drooling*; and VI, *Dry mouth*. The domains in the Examination part are: 1, *Face at rest*; 2, *Nose breathing*; 3, *Facial expression*; 4, *Masticatory muscles and jaw function*; 5, *Oral motor function*; and 6, *Speech*. The OB and NVR groups differ significantly with respect to domains II, V, and 1, 4, and 5, with a significance level set at *P* = 0.05 (marked with *).

The OFD profiles created from the NOT-S displayed similar trends (Fig. 2). The mean total NOT-S score was higher in the OB group (mean, 3.86 ± 2.46; median, 4; min–max, 0–10) than in the NVR group (mean, 3.16 ± 2.36; median, 3; min–max, 0–10), although the differences were not statistically significant.

The following NOT-S domains were significantly higher in the OB group than in the NVR: II, *Breathing*; V, *Drooling*; 1, *Face at rest*; 2, *Nose breathing*; IV, *Masticatory and jaw function*; and 5, *Oral motor function* (Fig. 2).

The most frequently contributing items in the OB group were: *Deviant lip posture*; *Deviant speech when counting to ten*; *Saliva leakage almost daily*; *Unable to bite hard on back teeth*; and *Snoring or sleep apnoea* (Table 2).

**Figure 3:**
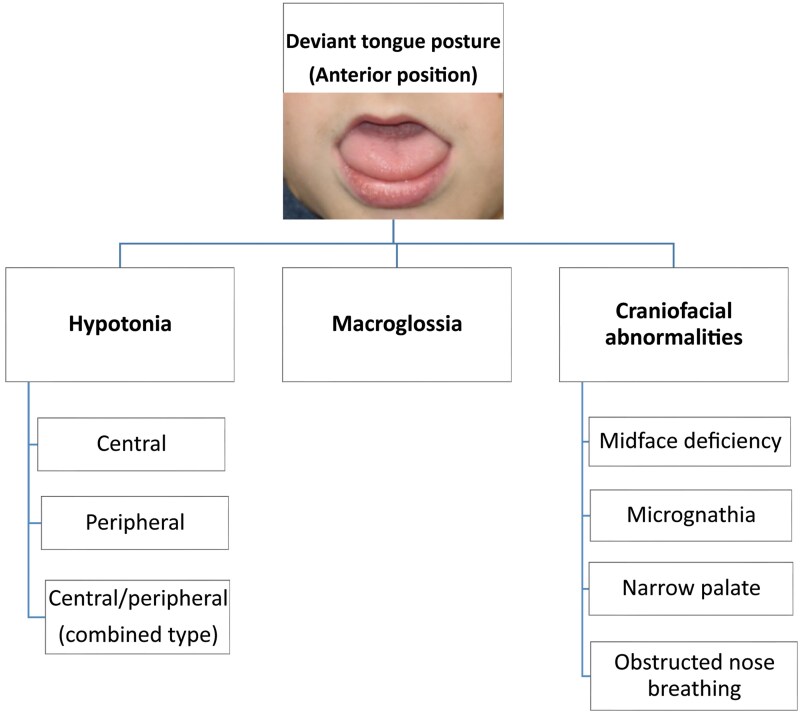
The figure shows the three main possible causes when a deviant tongue posture is present. Orofacial hypotonia is a common cause in individuals with rare diseases, although structural abnormalities, such as macroglossia, adeno-tonsillar hypertrophy or a mid-face deficiency, can also result in a deviant tongue posture.

The ORs for having an OB when scoring on different OFD items from the NOT-S were statistically significant for: *Deviant tongue posture*, *Breathing support*, *Unable to open mouth wide*, *Unable to breathe through nose*, *Saliva on chin daily*, and *Deviant lip posture* (Table 2). There were three symptoms that were associated with a deviant tongue posture in the sample (*n* = 238): orofacial hypotonia (48%), macroglossia (8%), and craniofacial or structural abnormalities (12%) (Fig. 3).

## Discussion

In this large sample of subjects with rare diseases, the OB prevalence was 18%, which is more than four-times higher than the prevalence of OB in Swedish school children. OB was a common finding, especially in individuals with neuromuscular diseases. Nemaline myopathy had the highest percentage of OB, including the most-severe sub-type (AOB_S_).

OB varied in terms of prevalence in the different age groups, in the range of 15%–25%. Some of the observed diseases and their orofacial symptoms are progressive, which suggests that OB may deteriorate with age. However, those groups with severe disabilities or progressive disease can have shorter life expectancies, which mean that the older age groups may comprise individuals with better health status and abilities. The prevalence of non-nutritive sucking habits was lower in this sample compared with a previous Swedish report investigating sucking habits in children with typical development in similar age group (4%–66%) [25]. The same study showed a significantly higher OR for developing AOB for those children who had or had had a sucking habit than for those who had never had a sucking habit. In this sample only 15% of those with a non-nutritive sucking habit age 3 to 6 years of age had an OB, which may suggest that there are other factors negatively influencing occlusal development in this patient group.

The participants in this study presented a wide range of total NOT-S scores. Rare diseases are serious, often chronic, and sometimes progressive conditions, and the symptoms vary between the diseases and between individuals with the same diagnosis. In this sample, almost two-thirds of the individuals showed an OFD, which underlines the importance of early assessment and targeted interventions to sustain or improve function. Some disease groups with specific symptoms seem to have a higher prevalence of OB. The NOT-S profiles were similar between the groups, although some of the domain scores showed a statistically significant difference. Importantly, orofacial dysfunction was also highly prevalent among individuals with normal vertical relation, and the differences between groups were mainly confined to specific NOT-S domains rather than the total score. In addition, the NOT-S revealed a 6-fold increase in the odds of having OB when a deviant tongue posture was present, emphasizing the potential importance of tongue posture in occlusal development. There are many factors that may contribute to OB, however when an open bite is establish it is inevitable that the tongue will be positioned in the opening, maintaining or even worsening the vertical malocclusion. Lip seal is required for swallowing but in case of incompetent closure and an AOB the tongue will serve as the functional sealant by pressing towards the anterior teeth. This has been proposed as one of the primary causes of open bite development in several reports, but it has also been questioned due to the short duration of tongue pressure [10, 26, 27].

**Figure 4:**
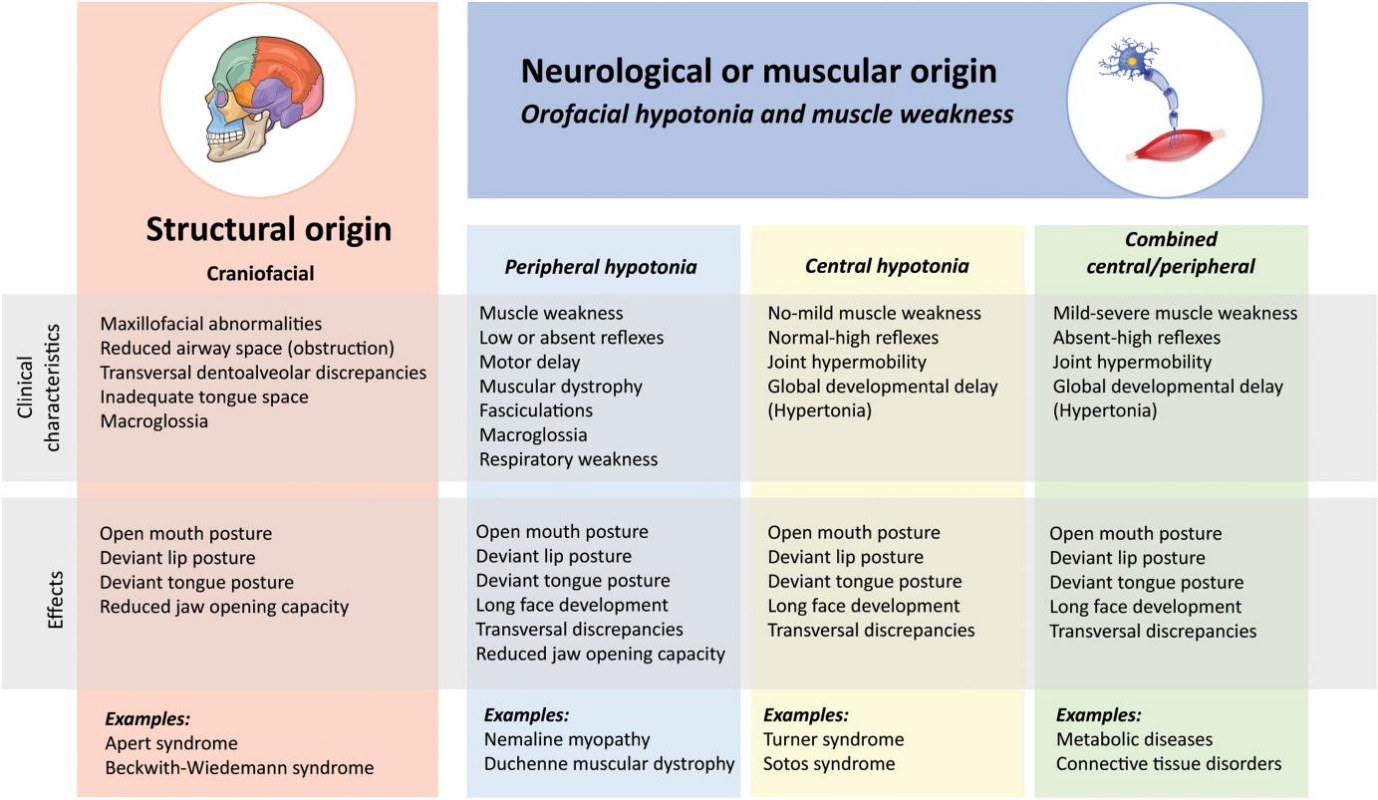
Schematic view describing two main disease groups that emerged from the sample that have clinical orofacial characteristics deriving from either a structural or neurological/muscular origin and their orofacial features and functions that are associated with open bite malocclusion. Illustration design: Fabbelito AB.

The intricate linkage between OB and OFD can be approached from different angles. There is a relationship between the form of a body part and its function, and it is said that the form always follows function. This could be interpreted as meaning that when a negative dentoalveolar development is present, it can be a sign of OFD. However, it is also claimed that form determines function, i.e. that the shape influences what the body part can do. This may mean that structural deficits in the orofacial area can inhibit or limit functionality. Rare diseases, although with different symptomatic origin might be more likely to present with OB development which is summarized in a schematic model (Fig. 4). When analysing different rare diseases there are three main symptoms that affect both form and function in the orofacial area and that seem to be associated with OB development: orofacial hypotonia, craniofacial abnormalities and macroglossia. These manifestations need to be discussed in relation to possible interventions.

### Orofacial hypotonia in neuromuscular and other genetic conditions

Low muscle tone or hypotonia is a symptom that appears in many diseases and syndromes. It is defined as residual tension in a muscle when it is resting. This low-level continuous contraction acts to maintain the posture and is, thus, important for orofacial function.

While muscle tone is not the same as strength, these features are often overlapping. Hypotonia is usually congenital and can originate from either the central nervous system (CNS, brain or spinal cord) or the peripheral nervous system (PNS, peripheral nerves, neuromuscular junctions, muscles). In general, central hypotonia often entails greater muscular strength and being hyperactive to normal reflexes, whereas in peripheral hypotonia the extent of muscle weakness is greater, and the reflexes are weak. The reduced ability of signals to reach the muscles can result in hypotonia, muscular atrophy and weakness. In the present study, many of the diseases with increased OB prevalence were associated with hypotonia of peripheral origin, with the subjects displaying OFD and muscle weakness. Open mouth posture is a common sign of hypotonia in individuals with OFD. *Face at rest* was the second-most-frequent domain in the OB group (50%; Table 2), and the items of *Deviant lip posture* (OR 1.422) and *Saliva on chin daily* were both associated with higher odds of having OB development (Table 2).

Neuromuscular diseases share certain symptoms. This means that there are different degrees of OFD, some with symptoms that increase in severity over time [28, 29]. Malocclusion is also a frequent finding in individuals with neuromuscular diseases [20, 21, 30–32], and they seem to be more prone to orthodontic treatment relapse [33]. Peripheral hypotonia and muscle weakness, as well as difficulties with opening the mouth widely are characteristic symptoms of neuromuscular diseases. Dysfunction of the masticatory muscle and jaw function were significantly different in prevalence between the OB and NVR groups, and both items were associated with OB. Although hypotonia itself cannot be resolved by voluntary control or exercise, there are indications that improving strength and endurance can have a positive impact on posture. In specific diseases with muscular dystrophy, special care should be taken to avoid causing fatigue or pain during orofacial exercises, so as to avoid muscle wastage. However, any form of exercise appears to be positive for most patients with neuromuscular diseases, although the evidence for a beneficial effect of strength training remains weak [34]. One study has specifically shown that for patients with progressive neuromuscular diseases, lip strength can be increased or sustained with training [35].

In most genetic conditions and in infants, the central or combined type of hypotonia is most common [36]. There are several rare diseases for which orofacial hypotonia with open mouth posture is the main characteristic, although the prevalence of OB is quite low. One example is Prader-Willi syndrome, in which the orofacial hypotonia is of mainly central origin and muscle strength is not as affected. Treatment of OFD related to hypotonia of central origin does not differ considerably from the treatment for hypotonia of peripheral origin. The choice of treatment should always be based on symptomatology and adapted to the individual’s abilities. Interventions that focus on improving the prerequisites for jaw, lip, and tongue posture and function, such as strength, stability, coordination, and sensory function, have clinical validity, and most studies have reported good results. However, most of the study groups to date have been too small and the design has been unsatisfactory [37].

Diseases that involve joint contractures are also associated to muscle weakness either as a primary cause or secondary to atrophy due to the restricted movement of the joint. This can be seen in individuals with arthrogryposis multiplex congenita [38], in whom OB is a common finding (Table 3).

### Structural abnormalities in craniofacial and growth-related conditions

Individuals with craniofacial syndromes can exhibit severe malocclusion due to deficient or abnormal craniofacial growth. OB are part of the orofacial phenotype in many of these cases, for example persons with Apert syndrome [39]. The skeletal deficiency can interfere with breathing and may impair masticatory function. Furthermore, these dysfunctions may negatively influence occlusal development by causing, for example, an open mouth posture. If present, the small mandible, maxilla and narrow palate will reduce the space for the tongue, resulting in an anterior tongue position, which can aggravate an OB. Severe OFD, such as feeding problems and sleep apnoea due to structural deficits, are often addressed at an early age, either with surgical interventions or compensatory strategies such as the use of ventilators. In the present study, the need for breathing support and the presence of apnoea were more common in the OB group according to the NOT-S. Breathing difficulty is a common problem in both neuromuscular diseases and craniofacial syndromes, albeit for different reasons (Fig. 4). The odds of having OB among individuals that were *Unable to breathe through the nose* (OR 2.017; Table 2) could be due to nasal obstruction or it could be linked to a habitual mouth breathing pattern. Nose breathing has long been discussed regarding OB and long face development [40–42], and it may be of greater importance when assessing individuals with disabilities. Obstructed nose breathing is strongly related to open mouth posture and can also aggravate problematic saliva leakage and speech resonance. If enlarged adenoids and tonsils are present, these can also cause dysphagia. A suspicion of obstructed nasal airways should always be assessed medically and followed up to avoid a mouth breathing pattern.

Several of the diseases with increased prevalence of OB, especially LOB, were associated with the presence of macroglossia. This is a rare orofacial symptom that is often difficult to differentiate from hypotonic tongue. Macroglossia has previously been reported in cases of Duchenne muscular dystrophy, Limb-Girdle muscular dystrophy, Beckwith-Wiedemann syndrome, and Turner syndrome [43]. It is also seen in acromegaly, and in tumour diseases, amyloidosis and oedemas that affect the tongue. In certain muscular dystrophies, the tongue becomes enlarged and wide at the base, which can affect the width of the dental arches, as well as the vertical relation. The development of macroglossia in muscular dystrophies is often seen in adolescence and follows the progression of the disease. The muscles are gradually replaced by connective and adipose tissue resulting in pseudohypertrophy of the tongue. In Beckwith-Wiedemann syndrome, the macroglossia is congenital and can cause problems with feeding and breathing in infants and young children. The macroglossia seen in Beckwith-Wiedemann syndrome does not increase in size, and indeed it can become less apparent over time. Surgical tongue reduction is sometimes performed in severe cases [44]. Deviant tongue posture showed the highest odds of OB (OR 6.062; Table 2): the criterion for that score was to be able to see at least the tip of the tongue between the teeth more than two-thirds of the time (test period: 1 min). This suggests that a deviant tongue posture (an anterior position of the tongue or tongue protrusion) may be highly influential in OB development, and that multiprofessional assessment and interventions are needed to improve orofacial function. A deviant tongue posture or severe macroglossia may need to be addressed at an early stage with sensory motor interventions or in rare cases, surgical tongue reduction.

## Clinical implications

- Severe anterior open bites are not exclusive to craniofacial syndromes but are also observed in patients with orofacial dysfunctions caused by neurological and/or muscular impairments.
- Early identification of potential underlying causes, together with aetiology-based prevention of orofacial dysfunction rather than solely symptomatic orthodontic treatment, may help reduce the risk of severe malocclusions and possibly limit relapse.
- Interdisciplinary collaboration may be valuable in both diagnosis and the implementation of complementary therapeutic strategies, addressing not only malocclusion but also associated orofacial dysfunction.
- A team with diverse expertise may provide more comprehensive support in complex cases and contribute to a more holistic, individualized approach, which might also reduce the future need for orthodontic treatment.

## Limitations

The study sample comprised of individuals who had been referred to Mun-H-Center orofacial resource centre for rare diseases or who had participated in the Ågrenska family programme. On the one hand, the sample has some selection bias, as most individuals who are referred to the clinic also have severe symptoms that need to be treated. On the other hand, those individuals with the most-severe intellectual or physical disabilities could not participate in the testing and could not be part of this sample. Therefore, individuals with either mild or severe symptoms risked exclusion from the study, which implies that the broader population may exhibit a greater range of function. However, the majority of individuals in the database were recruited through family stay programmes organized by a foundation, to which families applied independently, without necessarily having any known orofacial problems.

In this dataset, the overall proportion of missing data was moderate, with 11% missing values for the variable OB. Missing data were present across several variables, including other types of malocclusions, at even higher rates. As there was no clear pattern or known systematic cause behind the missingness, the data were assumed to be missing at random. However, this assumption should be interpreted with caution, as the possibility of nonrandom missingness cannot be entirely excluded.

A high number of subjects with the same disease may cause the results to become merely a description of the disease phenotype, rather than revealing the symptoms associated with OB. The largest disease group in the sample, Duchenne muscular dystrophy, has a high prevalence of OB and displays many of the risk factors that were identified in this study. For example, the respiratory musculature is often weakened in patients with DMD, requiring breathing support. It is possible that this finding is a co-existing phenotype description rather than a real association with OB. The item *Unable to open mouth wide* (OR 3.09) might also be a confounder, since some of the dystrophic myopathies also often have a reduced and deteriorating mouth opening capacity. However, there could be an association between OB and craniofacial abnormalities and the range and function of the temporomandibular joint.

One would expect that open bite would be more prevalent in the older age groups. However, it was difficult to analyse OB in relation to age, since some of the diagnoses with a high prevalence of OB are linked to a shorter life expectancy. There are also some milder forms of some diseases, as in myotonic dystrophy type 1, where symptoms can develop in adulthood. In addition, young individuals with severe OFD seemed to have developed an OB already early in life. If we had analysed the vertical measurements (in millimetres) instead of a nominal variable yes/no, we might have detected changes in prevalence in relation to age in more homogenous sub-groups of the sample.

## Conclusion

In this large sample of individuals with rare diseases, almost two-thirds displayed orofacial dysfunction, with open bite malocclusion being a frequent finding. Patients with diseases associated with orofacial hypotonia, macroglossia, and craniofacial abnormalities presented the highest proportion of open bite. A deviant tongue posture was identified as the having the strongest association with open bite based on the orofacial dysfunction test. Orofacial dysfunction was also common among individuals without open bite, underlining the need for functional assessment regardless of the presence of vertical malocclusion. These findings support the role of multiprofessional assessment in individuals with rare diseases, and point towards the potential value of early, interdisciplinary interventions with the aim of supporting function, occlusal development, and overall well-being. Future longitudinal and interventional studies are needed to clarify causal pathways and to determine the effectiveness of such approach.

## Supplementary Material

cjag037_Supplementary_Data

## Data Availability

The authors confirm that the data supporting the findings of this study are available within the article. The data will be shared upon reasonable request to the corresponding author.
