# Supplementary material for: Open bite malocclusion and orofacial dysfunction in patients with rare diseases
Source: Eur J Orthod. 2026 Jun 8;48(4):cjag037. doi: 10.1093/ejo/cjag037 (PMC13244795; doi:10.1093/ejo/cjag037)
Supplement: cjag037_Supplementary_Data [file cjag037_supplementary_data.zip › not_picture_manual.pdf]

NOT-S

Nordic Orofacial Test-Screening

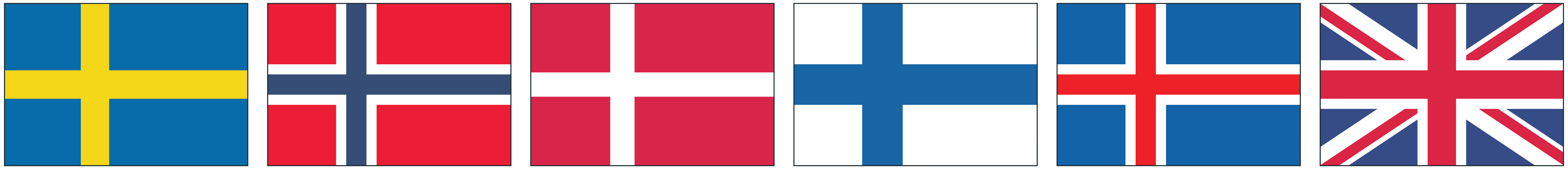

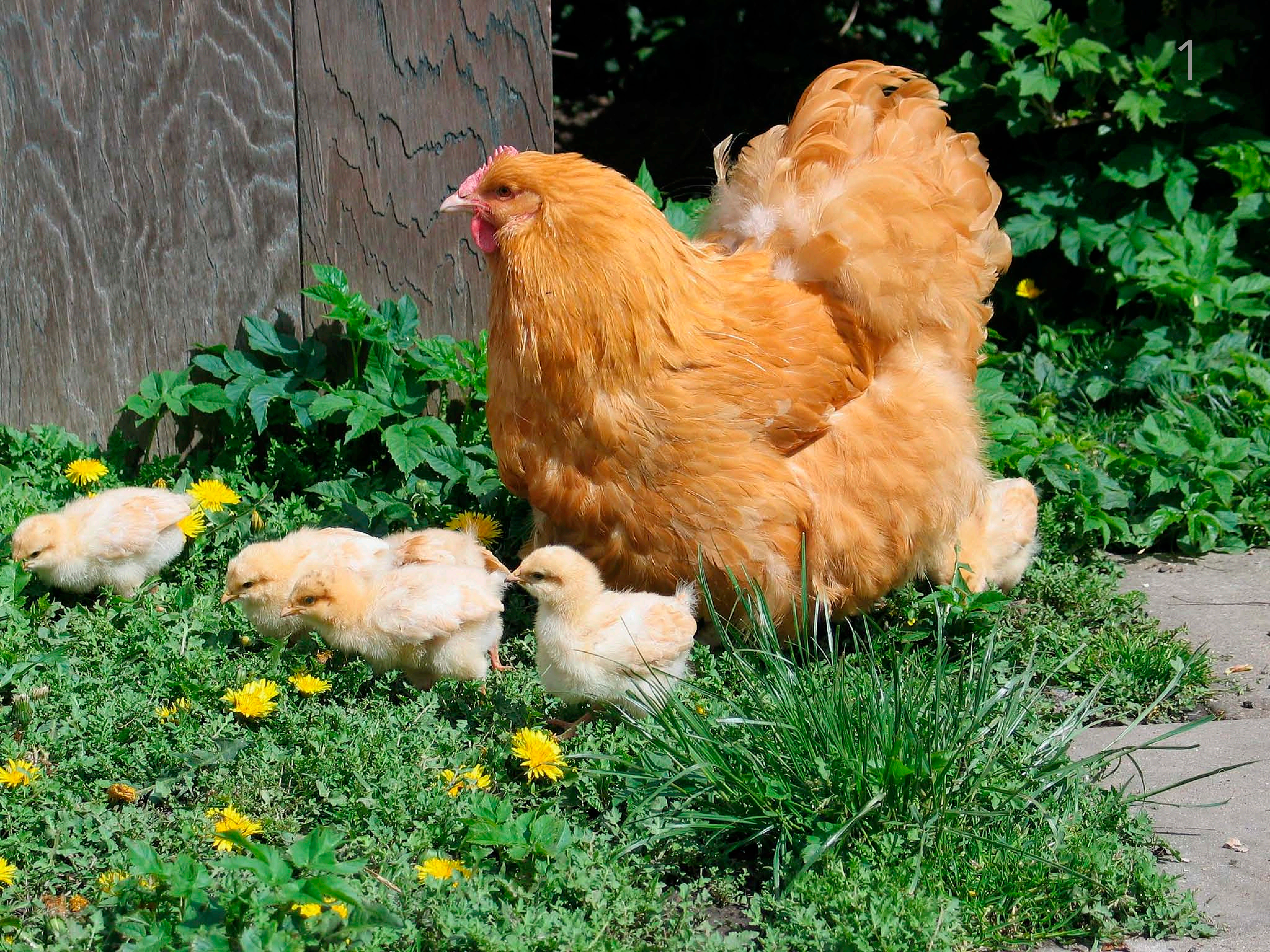

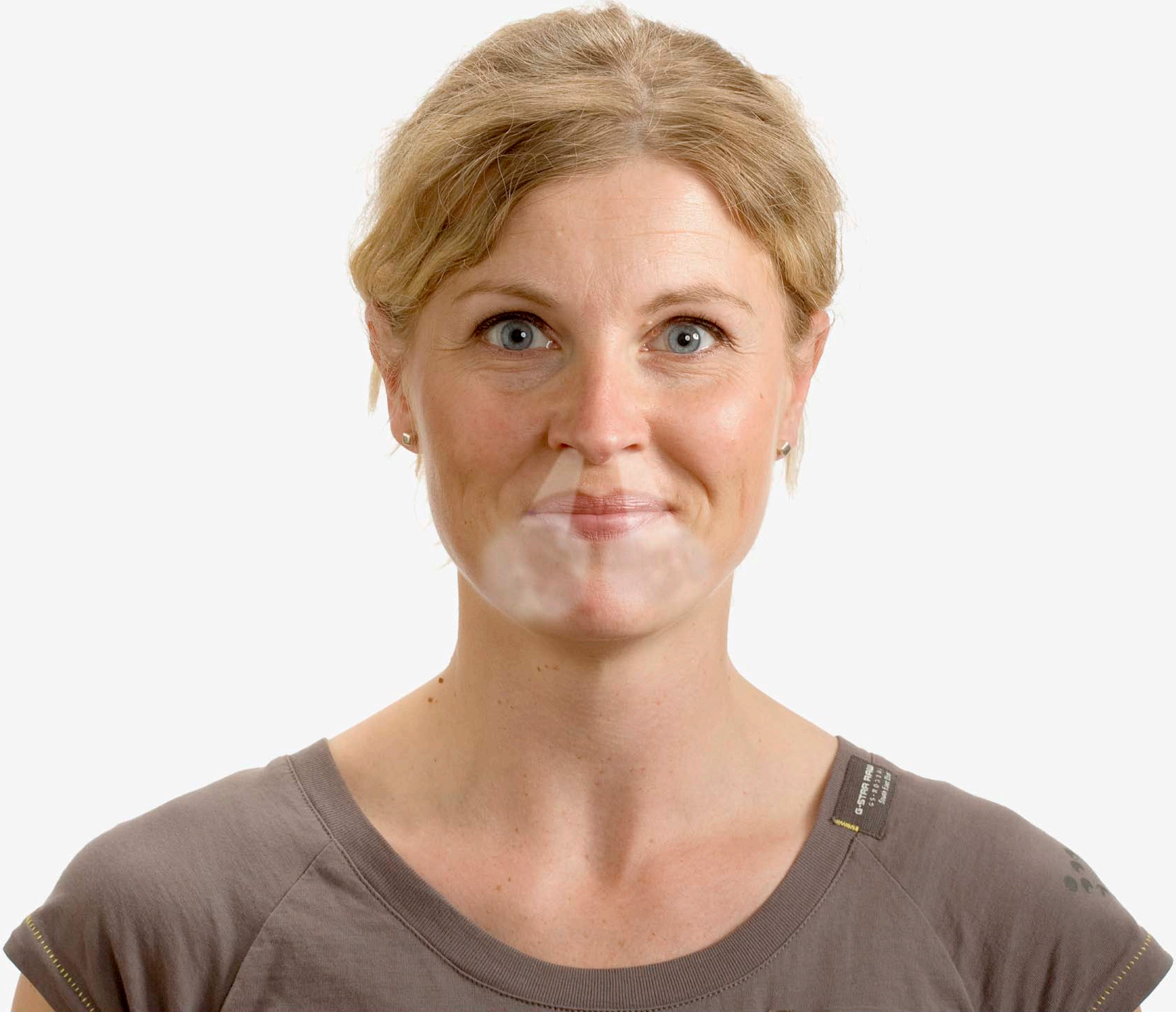

3A

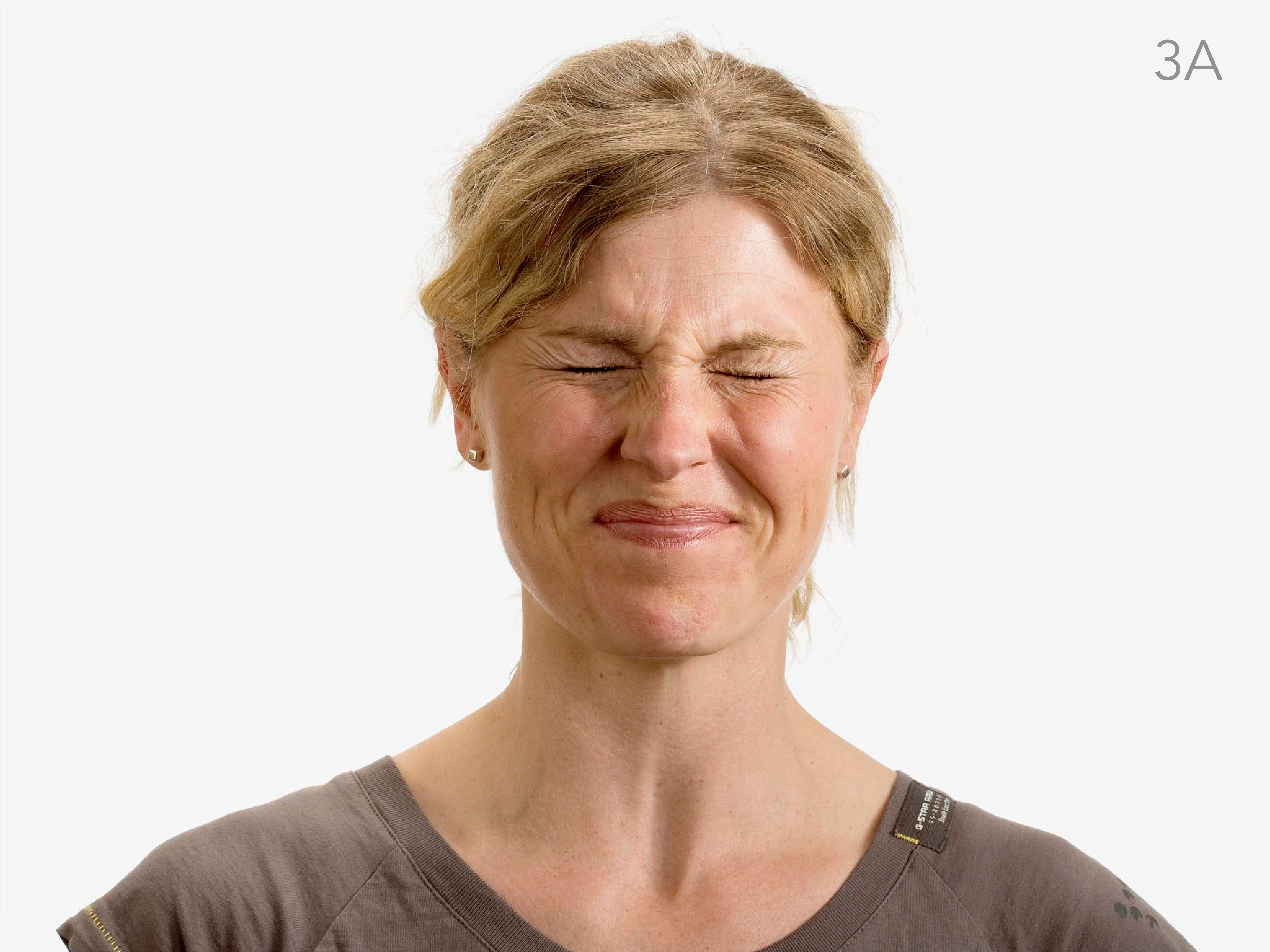

3B

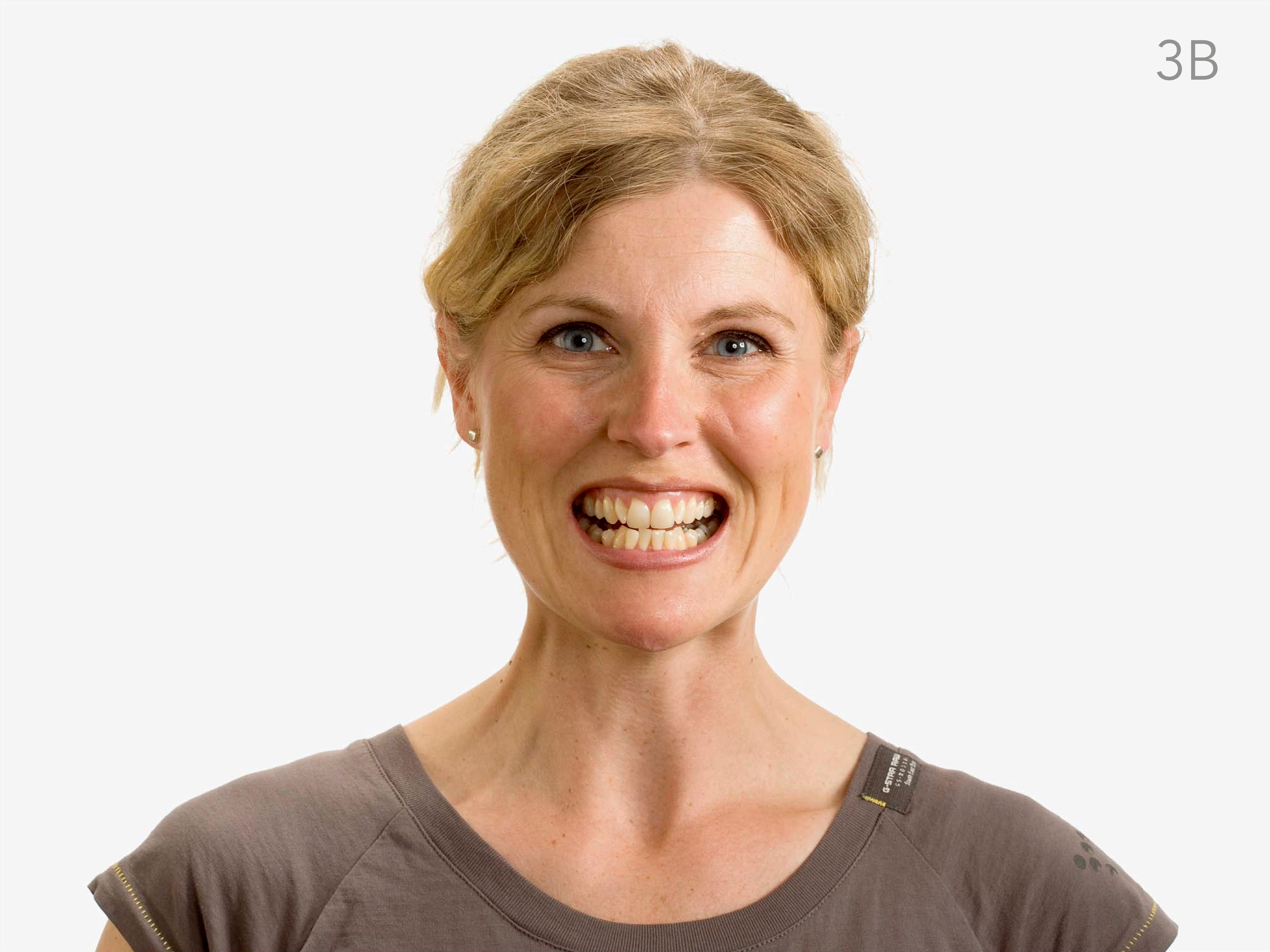

3C

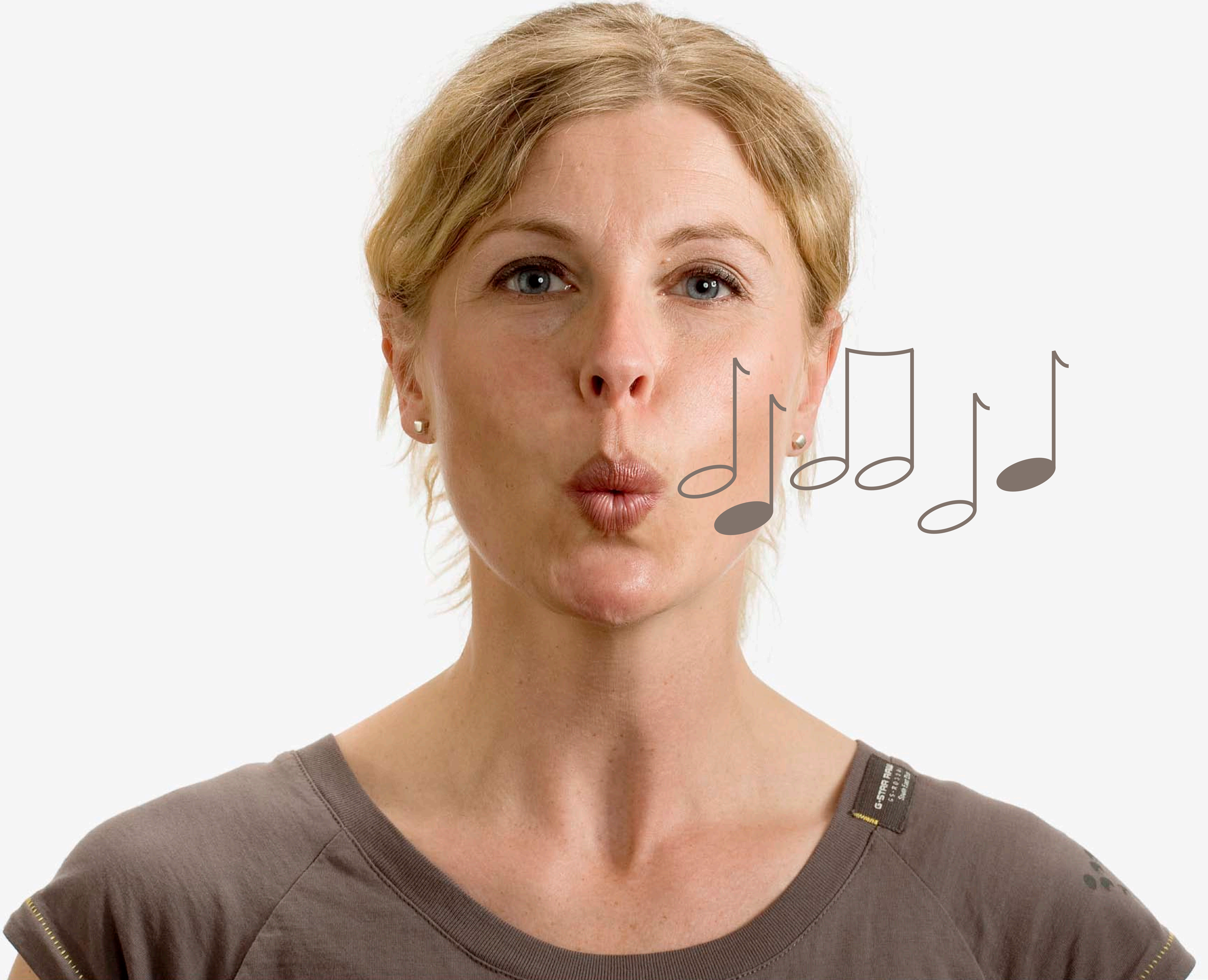

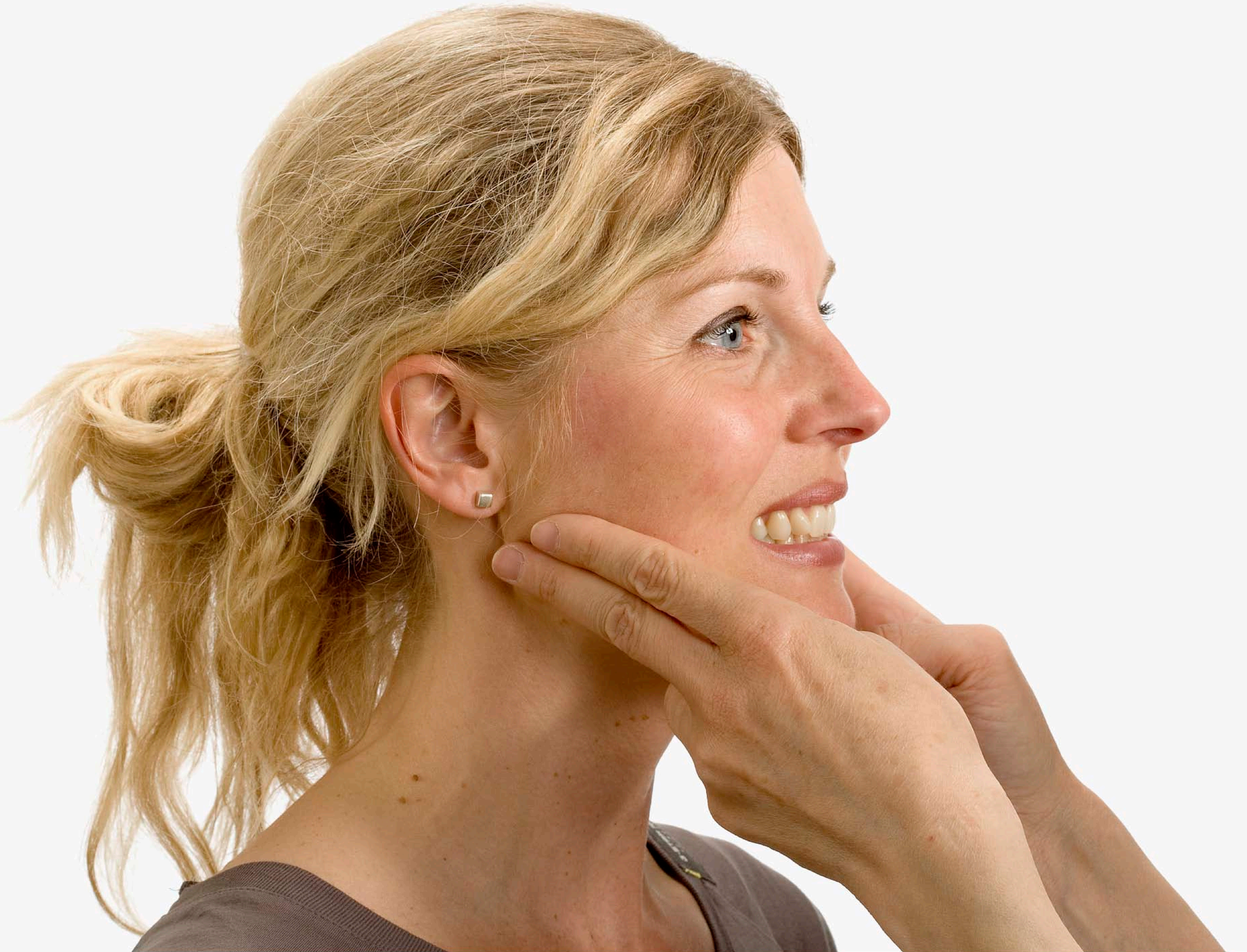

4A

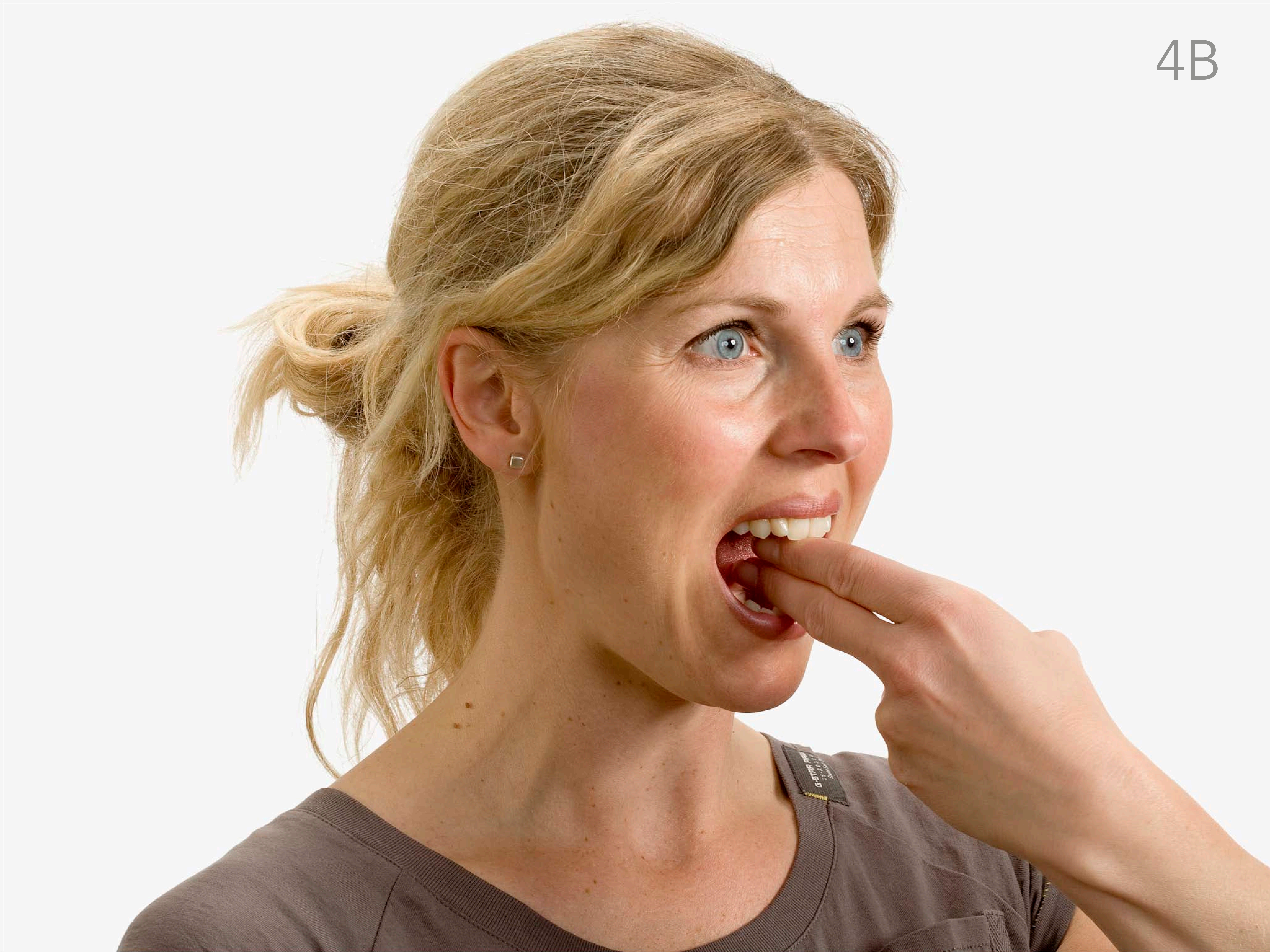

4B

5A

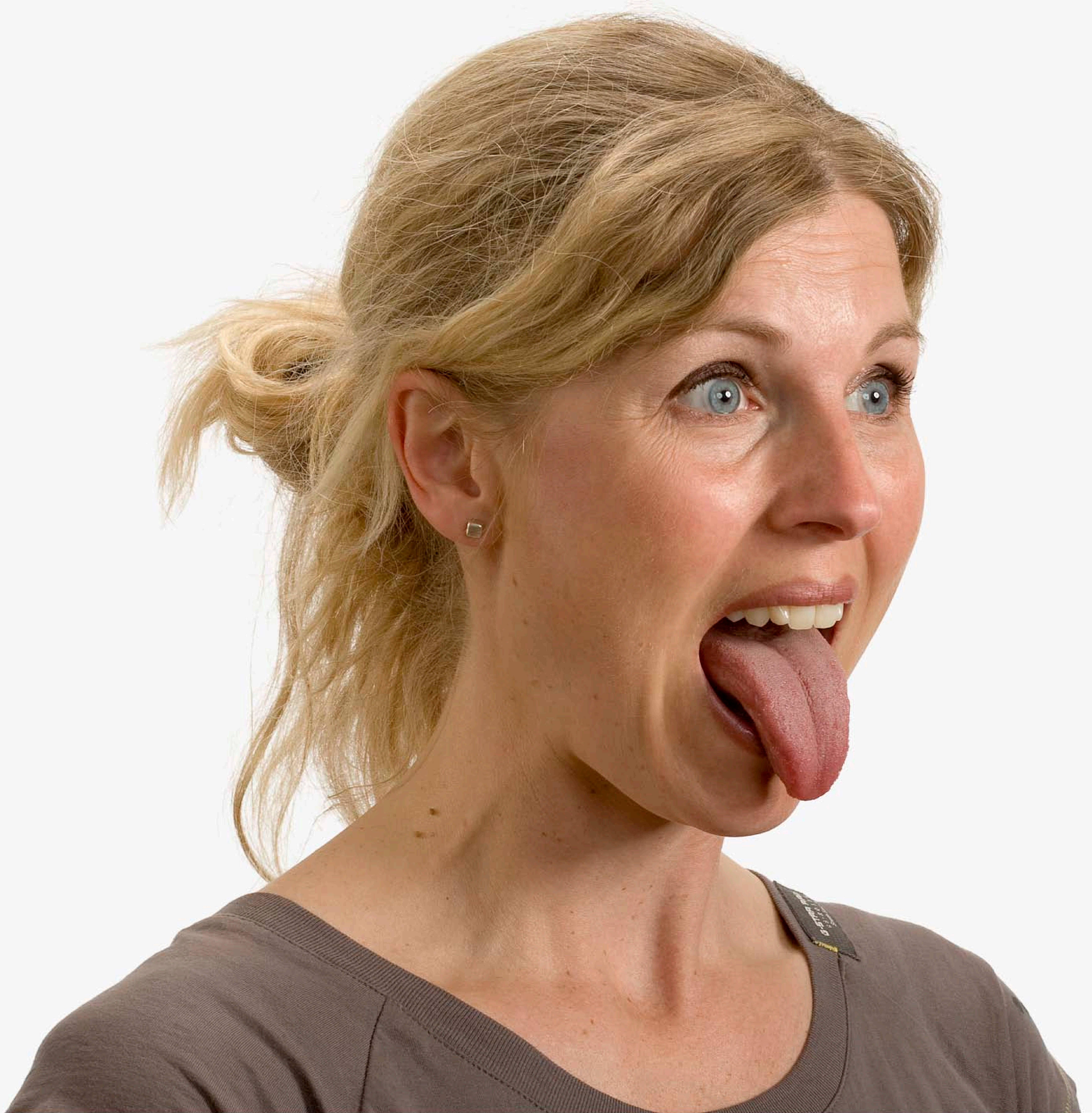

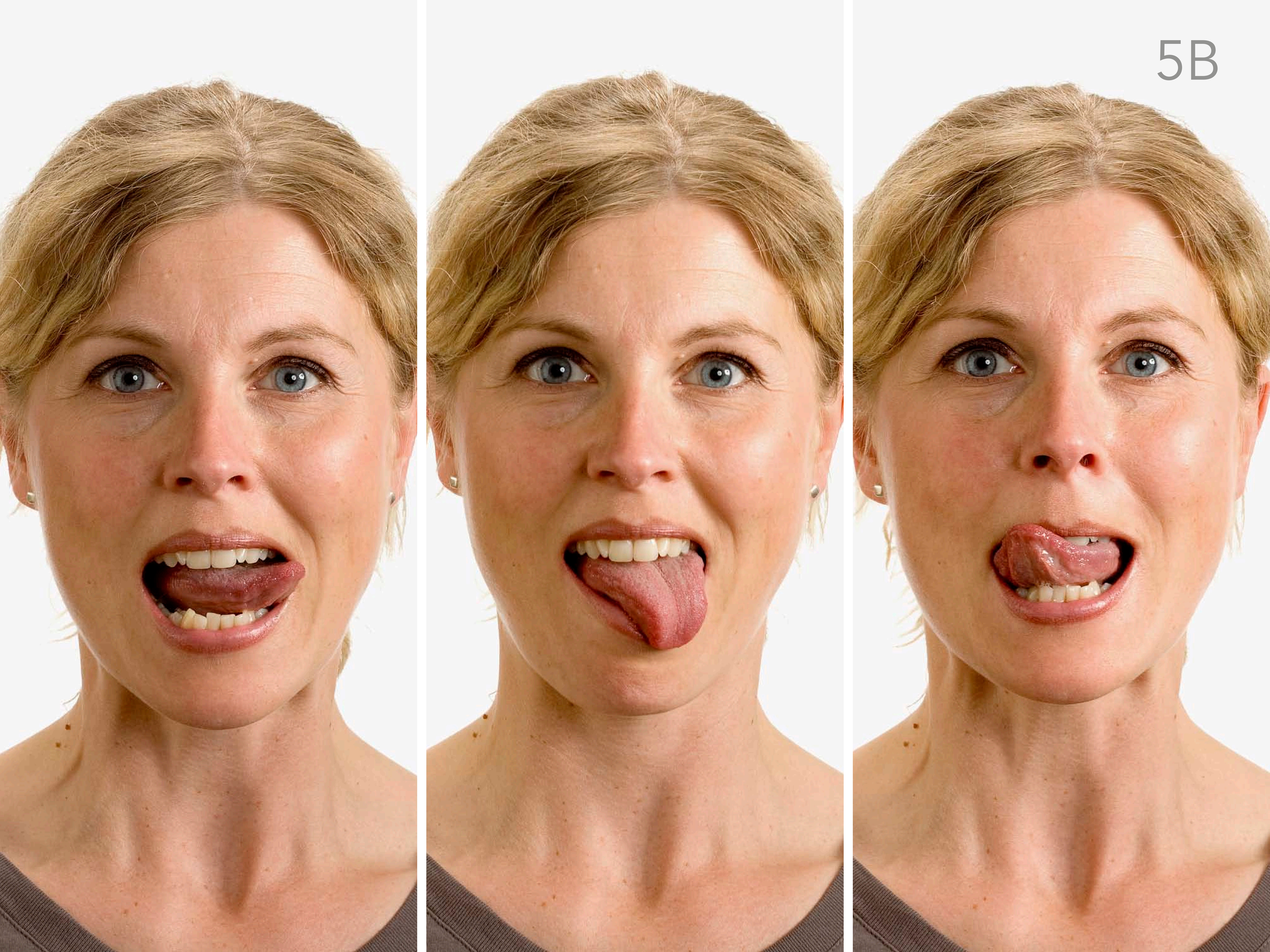
5B

5C

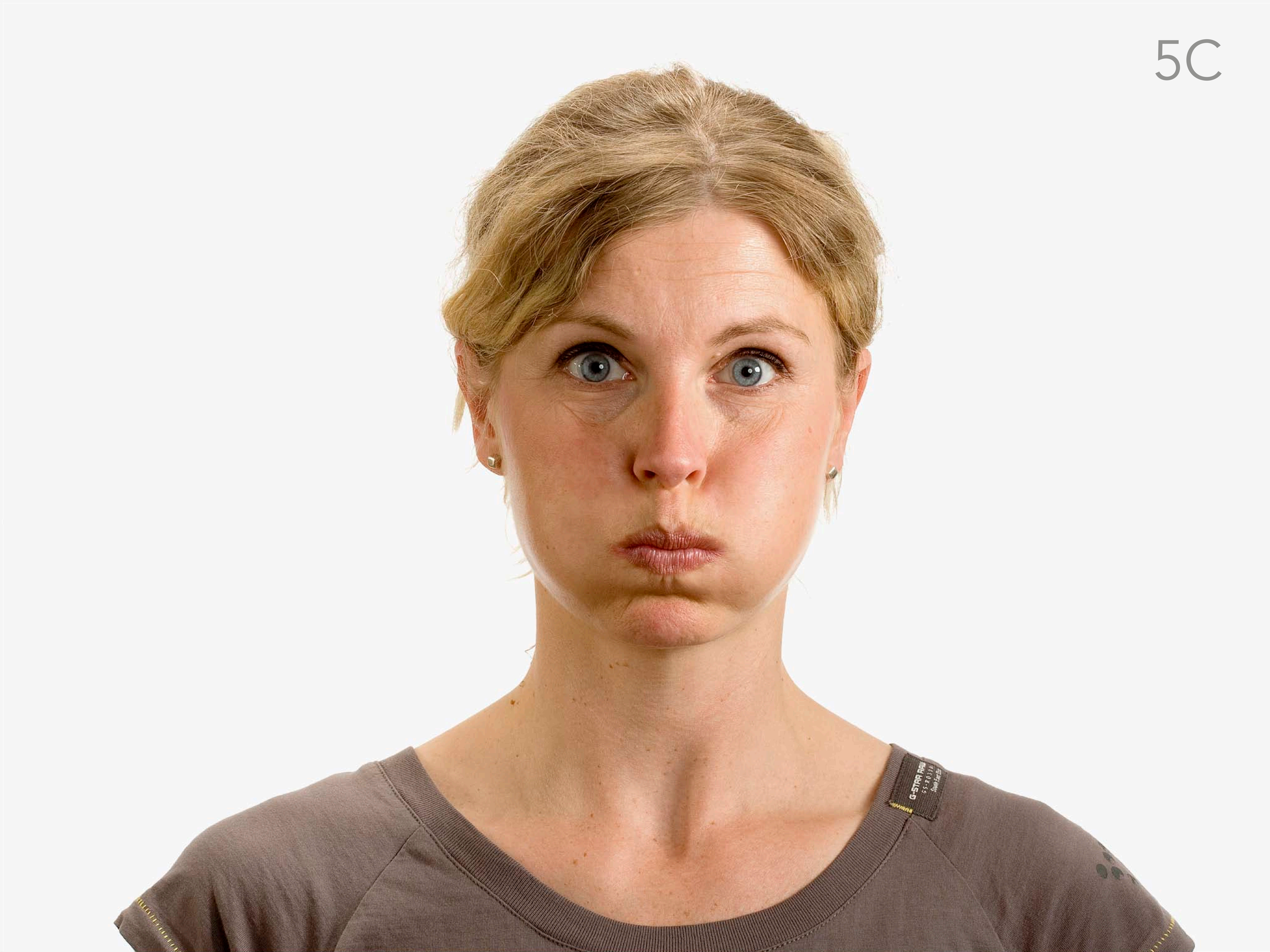

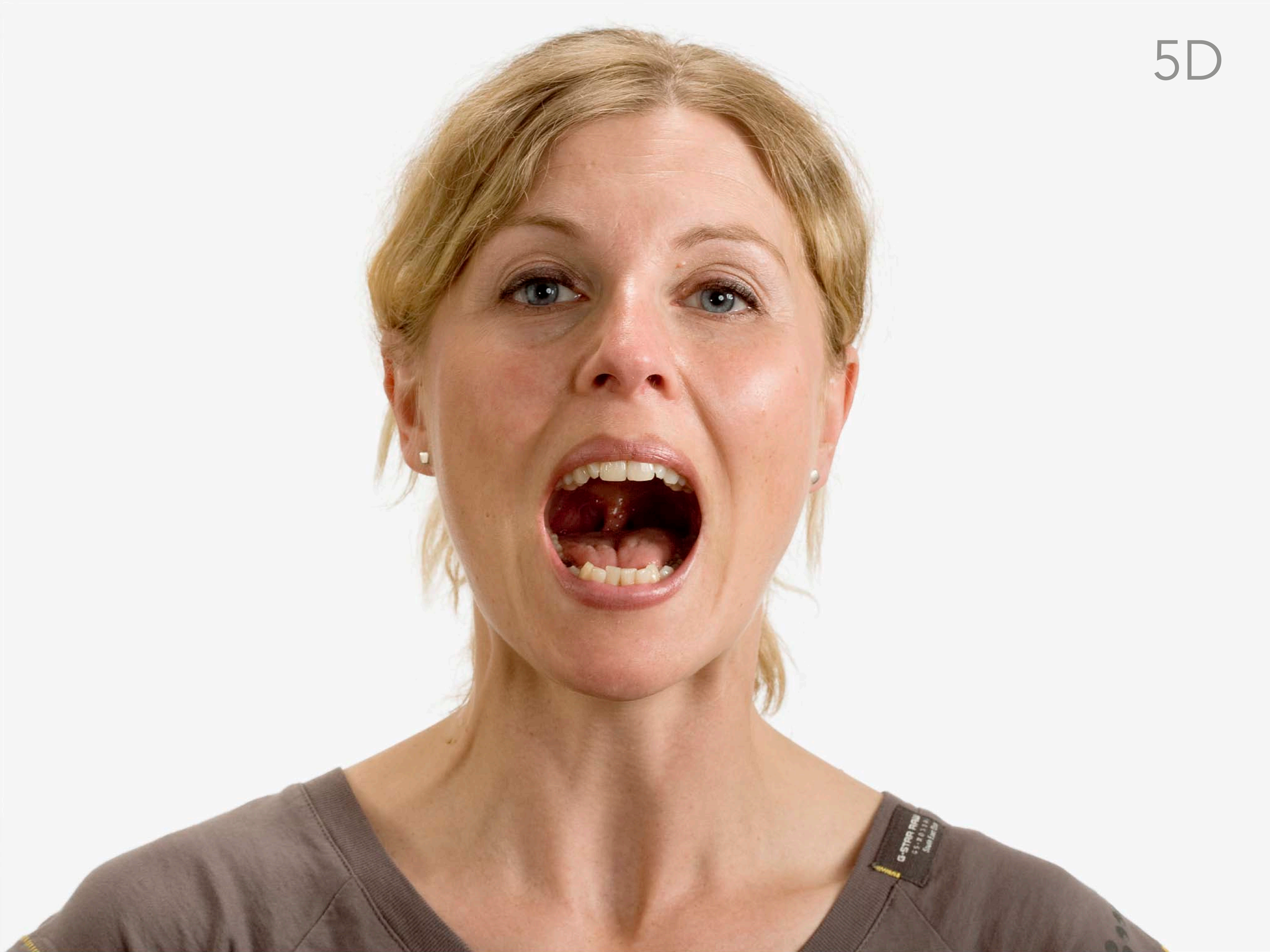

5D

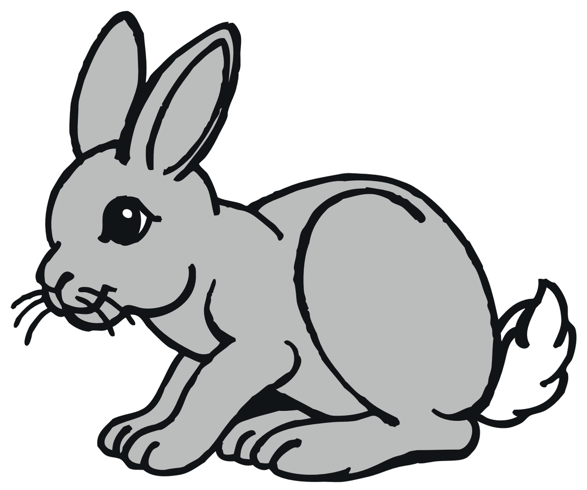
1

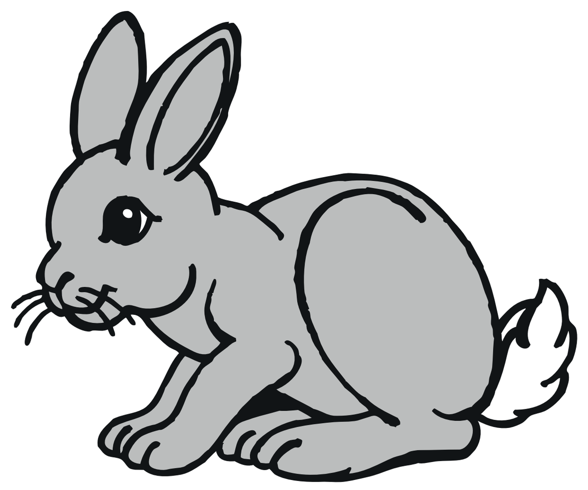
2

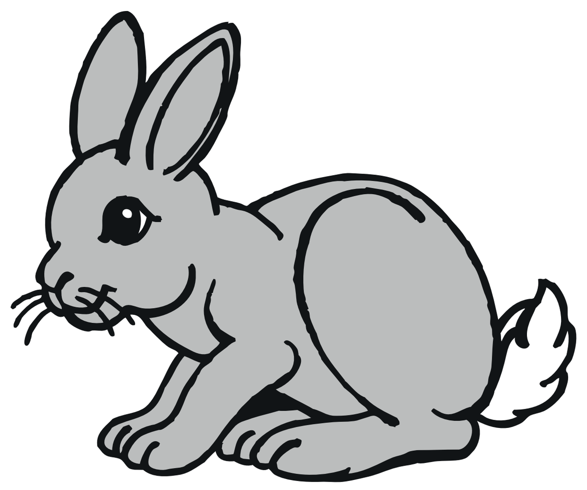
3

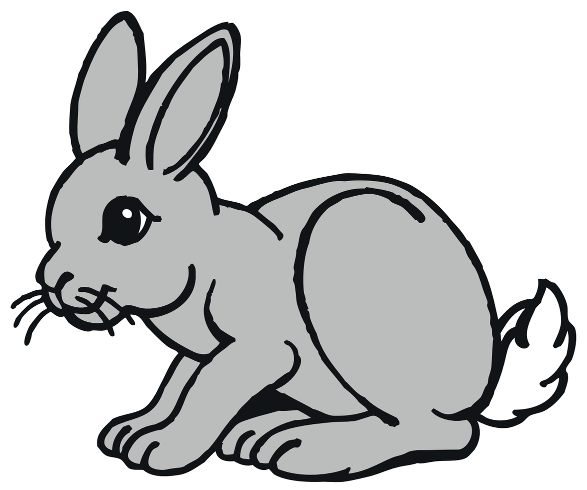
4

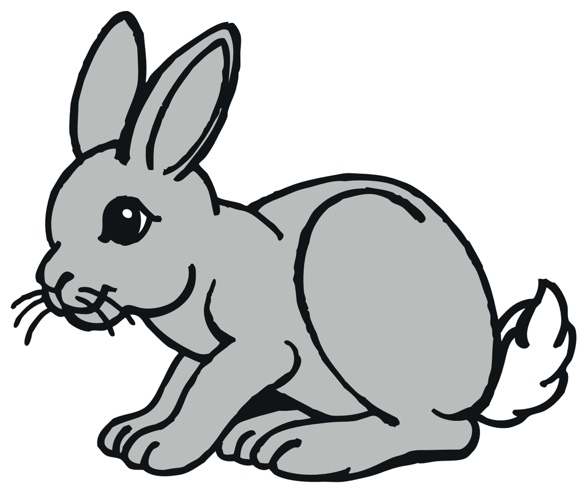
5

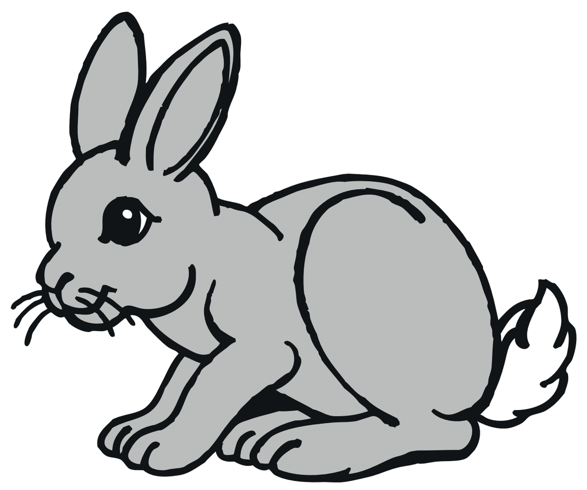
6

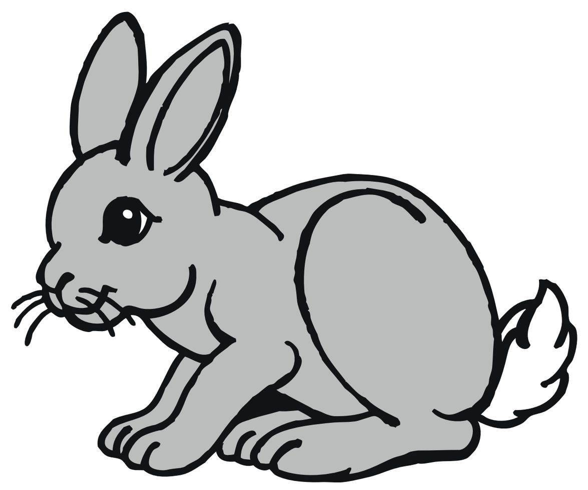
7

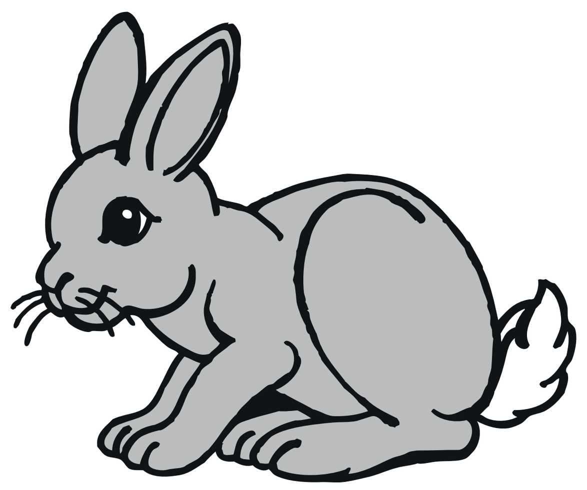
8

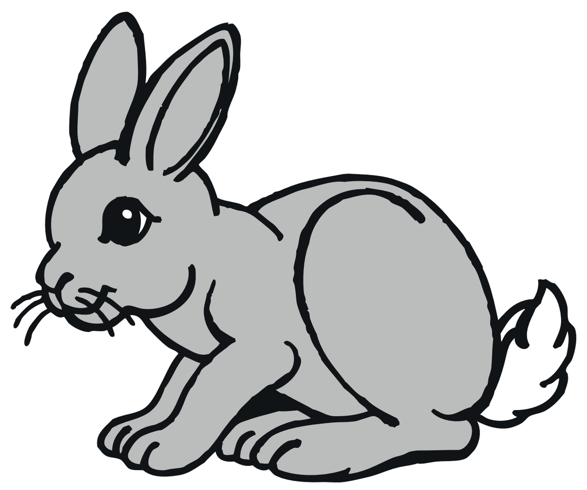
9

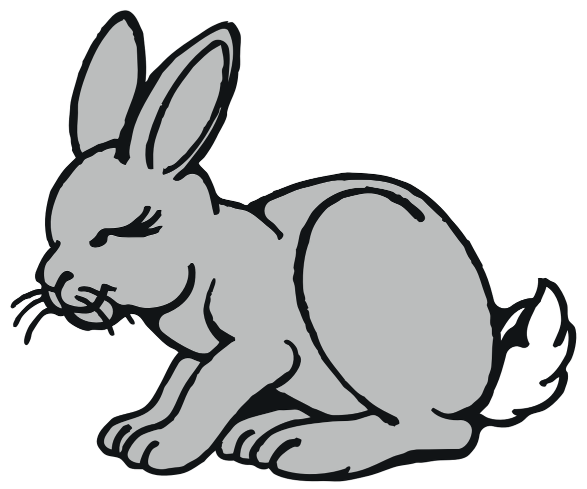
10

PA-TA-KA
PA-TA-K
PA-TA-KA

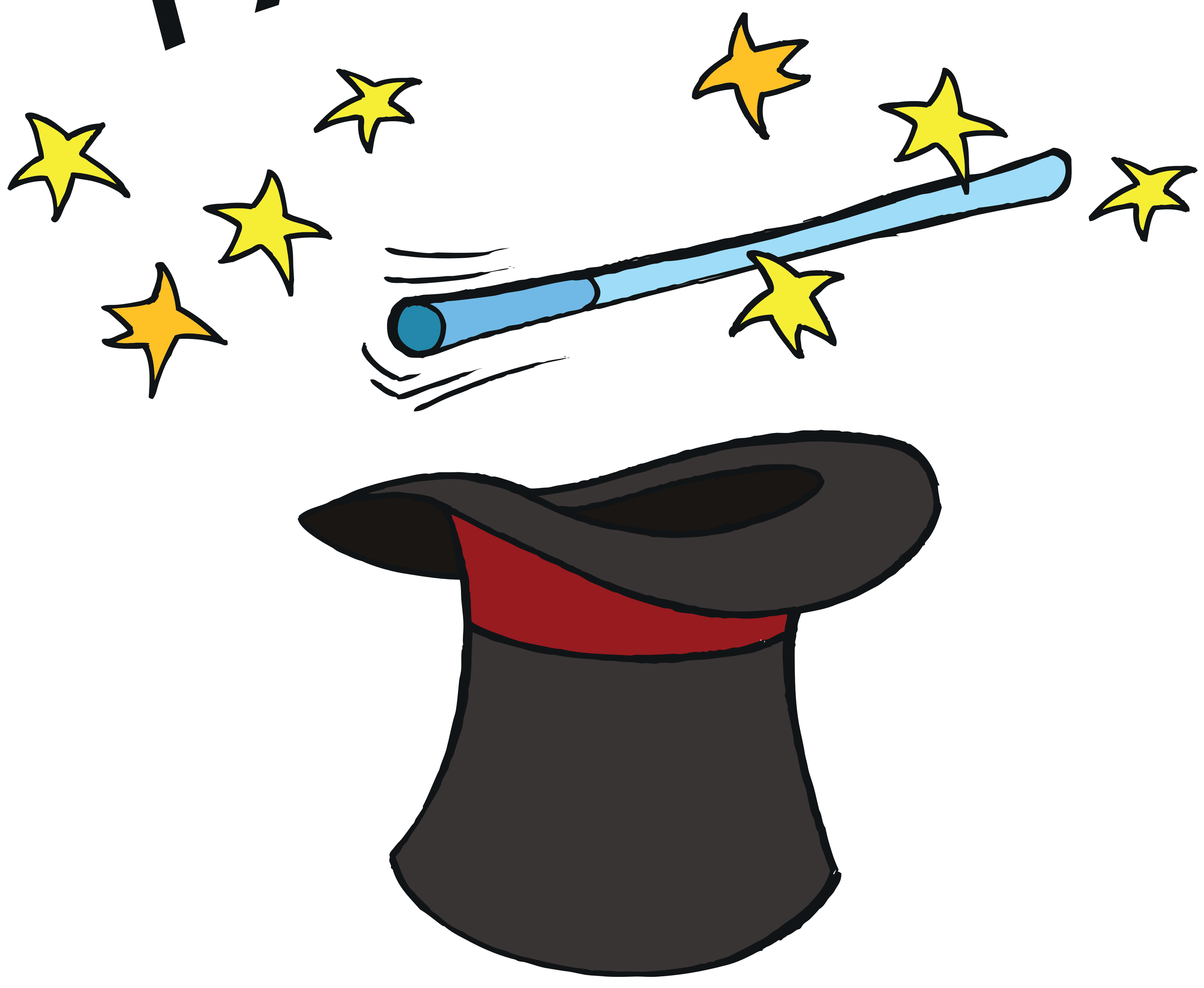

Nordic Orofacial Test-Screening, NOT-S

The examination form and the picture manual can be downloaded from:

www.mun-h-center.se

Mun-H-Center +46 10 441 79 810

NOT-S was developed with support from the Nordic Association for Disability and Oral Health, NFH

Working group
Merete Bakke, Copenhagen, Denmark
Birgitta Bergendal, Jönköping, Sweden
Anita McAllister, Linköping, Sweden
Lotta Sjögreen, Gothenburg, Sweden
Pamela Åsten, Oslo, Norway

2006 © NFH, Digital version 2017
